# Using Metabolomic Profiles as Biomarkers for Insulin Resistance in Childhood Obesity: A Systematic Review

**DOI:** 10.1155/2016/8160545

**Published:** 2016-07-19

**Authors:** Xue Zhao, Xiaokun Gang, Yujia Liu, Chenglin Sun, Qing Han, Guixia Wang

**Affiliations:** ^1^Department of Endocrinology and Metabolism, The First Hospital of Jilin University, Changchun 130021, China; ^2^Hospital of Orthopedics, The Second Hospital of Jilin University, Changchun 130021, China

## Abstract

A growing body of evidence has shown the intimate relationship between metabolomic profiles and insulin resistance (IR) in obese adults, while little is known about childhood obesity. In this review, we searched available papers addressing metabolomic profiles and IR in obese children from inception to February 2016 on MEDLINE, Web of Science, the Cochrane Library, ClinicalTrials.gov, and EMASE. HOMA-IR was applied as surrogate markers of IR and related metabolic disorders at both baseline and follow-up. To minimize selection bias, two investigators independently completed this work. After critical selection, 10 studies (including 2,673 participants) were eligible and evaluated by using QUADOMICS for quality assessment. Six of the 10 studies were classified as “high quality.” Then we generated all the metabolites identified in each study and found amino acid metabolism and lipid metabolism were the main affected metabolic pathways in obese children. Among identified metabolites, branched-chain amino acids (BCAAs), aromatic amino acids (AAAs), and acylcarnitines were reported to be associated with IR as biomarkers most frequently. Additionally, BCAAs and tyrosine seemed to be relevant to future metabolic risk in the long-term follow-up cohorts, emphasizing the importance of early diagnosis and prevention strategy. Because of limited scale and design heterogeneity of existing studies, future studies might focus on validating above findings in more large-scale and longitudinal studies with elaborate design.

## 1. Introduction

Obesity prevalence has nearly doubled worldwide during the past three decades and still continues to increase. Obesity during childhood and adolescence is reaching epidemic proportions with great increase in prevalence rate [1]. During 1971 to 1974, the prevalence rates of obesity in 6–11-year-old white/black children were 4% in the United States. Between 1999 and 2002, these prevalence rates increased to 13% and 20% in white and black children, respectively [2]. In 2012 the overall prevalence rate of obesity in 2~19-year-old American children was 17.3% [1]. In developing countries the prevalence rate of overweight and obesity in preschool children (<5 years old) in 2010 was estimated to be 6.1% and 11.7%, respectively [3]. Thus, the prevalence of childhood obesity has attracted wide attention of researchers and become an emerging problem for public health. Furthermore, a variety of evidences have shown the close relationship between childhood obesity and multiple serious health complications with high risk of morbidity and mortality [4, 5].

Insulin resistance (IR), contrary to insulin sensitivity (IS), refers to decreased response to insulin-mediated cellular actions [6]. In particular, it can be considered as impairment in the function of insulin to induce glucose uptake in targeted tissues and to suppress production and output of hepatic glucose [7]. Furthermore, it is indeed associated with a resistance to insulin action on protein and lipid metabolism and on vascular endothelial function and genes expression [8]. IR is the most common metabolic alteration related to obesity [9], and it represents an important link between obesity and type 2 diabetes [10] and other metabolic risks such as cardiovascular diseases [11, 12]. Thus, identifying children with IR could be an effective strategy for the prevention and treatment of obesity related complications. Despite the validity of gold-standard hyperinsulinemic-euglycemic clamp and frequently sampled IV glucose tolerance test (FSIVGTT) [13], their costly and time- and labor-intensive features limit the applications in large epidemiologic studies [14]. Homeostasis model assessment for insulin resistance (HOMA-IR) provides an estimate of IR derived from fasting glucose and insulin levels, with higher scores representing a greater degree of IR. It has been validated as a surrogate marker of IR for clinical and epidemiological studies of children and adolescents, while HOMA-IR still has its limitation in early diagnosis of IR in order to prevent its progression to type 2 diabetes and other metabolic disorders. Thus, more sensitive and specific biomarkers are urgently needed to detect IR as soon as possible.

Metabolomics is a technique for identifying and quantifying endogenous small molecule metabolites (<1,500 Da) [15]. A wide range of metabolites in blood, urine, or tissues can be detected by the application of nuclear magnetic resonance spectroscopy (NMR), liquid or gas chromatography-mass spectrometry (LC-MS, GC-MS), and so on [16]. Due to its unique advantage in reflecting minimal metabolic alteration, applying metabolomics as biomarkers in clinical studies has generated more and more speculations. Our previous work showed metabolomics profiles can be useful biomarkers and predictors for diabetic kidney disease (DKD) diagnosis and its progression [17]. Recent studies on obese adults have presented the alterations in metabolomics not only reflect metabolic disorders, but also shed light on the fundamental mechanism of diseases [18, 19]. In the last 5 years, studies focusing on the relationship between metabolomics and IR in obese children have emerged quickly, while results seem to be controversial. Thus, our aims were to integrate available IR related plasma metabolomic profiles in obese children and find out specific biomarkers for IR and future metabolic risk.

## 2. Method

### 2.1. Literature Search

We did the systematic search for papers on MEDLINE, Web of Science, the Cochrane Library, ClinicalTrials.gov, and EMASE for relevant studies from inception to February 2016. A search strategy was applied based on medical subject headings (MeSH®) terms. To search relevant papers, variant combinations of following terms were applied: “childhood obesity”, “obese children”, “pediatric obesity”, “insulin resistance”, “metabolomic profiles”, “metabolomics”, “metabolic signature”, “metabolomic approach”, “metabolites”, “nuclear magnetic spectroscopy,” and “mass spectrometry”. In this procedure, two investigators (Xue Zhao and Qing Han) independently completed this work in order to minimize selection bias. If there were disagreements, a third investigator (G. Wang) will join in the selection procedure to solve the problem.

### 2.2. Inclusion and Exclusion Criteria

After reading all full-text articles, we decide to include or exclude them based on the criteria. The following inclusion criteria were applied: (1) all subjects should be children or adolescence, which means age of participants is <18 years; (2) obese children were defined as those with a body mass index (BMI; kg/m^2^) greater than the 95th percentile (≥95th) for age and gender according to the Centers for Disease Control growth reference [30]; (3) participants should be free of any thyroid or metabolic disorders requiring treatment such as diabetes, hypertension, severe dyslipidemia, and coronary heart disease; (4) studies need to include adiposity measures (BMI or BMI *z* score) and HOMA-IR according to the formula glucose (mmol/L) × insulin (mIU/L)/22.5 [31]; (5) metabolomic techniques such as MS, NMR spectroscopy, or UPLC-MS were applied to detect metabolite profiles in blood and results of plasma global metabolomics should be the outcome; (6) only papers published in English were included in this review.

Studies were excluded if subjects had diabetes or if they were >18 years old or if they had severe medication history. The metabolomics profiles extracted from urine are ruled out. Conference abstracts, reviews, meta-analyses, case reports, and letters to the editor were also excluded.

### 2.3. Data Extraction and Analysis

Data extraction on characteristics of study population, HOMA-IR, and identified metabolomic profiles were extracted, the procedure which was done by two different people (Xue Zhao and Qing Han). Due to large heterogeneity in study designs, method, and population characteristics, we did not apply a quantitative meta-analysis.

### 2.4. Methodological Quality Assessment

QUADOMICS was used to evaluate the included studies in methodological quality. It was developed to assess quality issues specific to “omics” research, including the quality assessment of studies included in systematic reviews [32]. The results after QUADOMICS were “high quality” or “low quality,” which refer to the methodologies of studies that achieved 11/16 or lower, respectively.

## 3. Results

### 3.1. Literature Search and Study Characteristics

**Figure 1:**
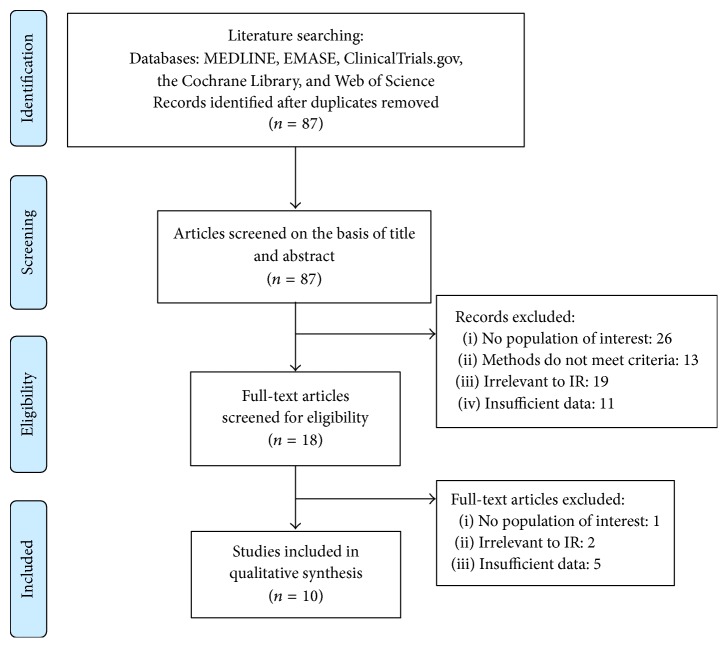
The flowchart of literature selection in this review.

We include 10 studies (including 2,673 participants) after systematic selection. All included studies met the inclusion/exclusion criteria. We performed literature selection according to PRISMA statement [33] in Figure 1. After literature research, 87 potentially relevant papers were firstly identified, among which 18 full-text articles were retrieved. Finally, 10 unique studies were included in our systematic review finally, which provided adequate data and information.

We extracted population characteristics of included 10 studies in Table 1, including the year, sample size, boy/girl proportion, study type, mean age, BMI *z* score/BMI, HOMA-IR, and identified metabolomics profiles. All the ten studies were published between 2012 and 2016. Eight studies were conducted in the United States [21, 23–29], one in Korean [22], and one in Germany [20]. The studies varied in sample size from 69 to 984 with a median of 267. The race/ethnicity of participants was various, including Hispanic, African American, White, and Asian. Nine studies included both boys and girls; only one study focused on boys [22]. Two studies mentioned sex difference in metabolomics profiles in obese children [24, 26]. Mean baseline ages of participants ranged from 4 to 19 years, with a median of 11.1 ± 1.3 years. Seven of ten studies belong to observational study; the remainders were cohort studies with 12-month, 18-month, and 24-month follow-up, respectively [20, 22, 26]. The analytical platforms used for metabolites detection included liquid chromatography-mass spectrometry (LC-MS), gas chromatography-mass spectrometry (GC-MS), flow injection tandem mass spectrometer (FIA-MS/MS), nuclear magnetic resonance spectroscopy (NMR), HPLC/fluorescence spectroscopy, and ultra-performance LC-MS.

### 3.2. Extraction of Specific Metabolomics Related to IR and Future Metabolic Risk

After summarizing the metabolomics profiles in Table 1, then we divided them into several subgroups according to their related metabolic pathways and extracted specific metabolomics found to be associated with IR which was shown in Table 2. Metabolisms of amino acids and lipids were found to be most relevant to IR in obese children. In these two metabolic pathways, BCAAs (valine, leucine, and isoleucine), aromatic amino acids (tyrosine), and acylcarnitines (C3, C5) were the most frequently mentioned metabolites as novel biomarkers for IR. In three cohort studies with long-term follow-up, BCAAs and tyrosine were also reported to be good biomarkers for future IR and metabolic risk.

### 3.3. Quality Assessment

According to QUADOMICS, we conducted quality assessment process [32, 34]. The results showed four of the ten studies were classified as “low quality.” The remaining six studies were classified as “high quality.” Two different persons checked the general characteristics selection and methodological quality assessment independently.

## 4. Discussion

To the best of our knowledge, this is the first systematic review summarizing all available studies focusing on metabolomics and IR in childhood obesity. Comparing with nonobese children, obese children presented distinct metabolic patterns in metabolomics profiles [24]. Although available studies in this area are largely lacking, eligible studies still shed light on the novel features of metabolomics profiles in reflecting and predicting IR related metabolic risk, which might be beneficial to future clinical early diagnosis and treatment [35].

### 4.1. Metabolomics as Biomarker for IR in Obese Children

#### 4.1.1. Metabolomics in Amino Acids Metabolism

*BCAAs*. BCAAs, including valine, leucine, and isoleucine, are essential amino acids changing along with the consumption of a protein-containing meal [18]. Among a wide range of metabolites in blood, BCAAs have been suggested to play key roles in obesity, IR, and diabetes [15–17]. Studies on metabolomics approaches reported that insulin resistant rats and adults had an increased level of circulating BCAAs and their related metabolites [16, 19]. In the study on 803 Hispanic adolescents of Butte et al. [21], significant increase was seen in BCAAs and their catabolites in obese children which made the largest contribution to BMI and adiposity among global metabolomics. And elevated BCAAs were closely related to total energy expenditure (TEE) and IR. Perng et al. [23] also manifested the similar results in 262 children (6–10 years). They found that Factor 4 (characterized by BCAA related pattern) was higher in obese children than their counterparts. This increase was associated with adiposity and worse cardiometabolic factors including HOMA-IR. Each increment in the BCAA corresponded with 6% (95% CI: 1, 13%) higher HOMA-IR. In addition, children born to obese women (41.2%) had 0.61 (0.13, 1.08) higher BCAA score comparing with their counterparts, which suggested maternal obesity might also play a role in altered offspring BCAAs metabolism. Newbern et al. [24] revealed the sex differences in metabolomics profiles related to IR in 82 obese adolescents. They found male had higher BCAA related metabolites than female. Furthermore, boys' HOMA-IR correlated positively with BMI *z* score, elevated BCAAs, and uric acid, while in girls, HOMA-IR only correlated with BMI *z* score. Thus, recent studies reported the positive association between elevated BCAA and IR, which was mainly consistent with the results in adults while it also indicated that alterations in BCAAs metabolism could appear in early life, which might make major contribution to later metabolic disorders in life if no effective preventive strategy was performed.

However, inconsistent results were reported by other two previous cross-sectional investigations on adolescents. In the study by Mihalik et al. [28] and Michaliszyn et al. [27], obese adolescents did not show higher BCAA concentrations compared with normal weight counterparts. Furthermore, study by Michaliszyn et al. [27] showed BCAA levels were positively correlated with *β*-cell function relative to insulin sensitivity in a total of 139 obese and normal weight children as a result of adaptive metabolic plasticity in early life, while after generating available recent studies, this inconsistency could also be attributed to subject and method heterogeneity and differences in energy expenditure between subjects. In these two studies, the participants were divided into two obese subgroups, with and without dysglycemia and 17 patients with diabetes were included in dysglycemia group. The inclusion of patients who had already progressed into diabetes might influence their results. Furthermore, they did not analyze the results by principal components analysis (PCA), an unsupervised linear mixture model aimed at accounting for the variance within a dataset by a smaller number of mutually uncorrelated PCs, and they also ignored the possible variance between male and female in metabolomics profiles. In addition, subjects in subgroups showed different energy expenditure of 1507 kcal/24 h (normal weight), 1978 kcal/24 h (obese without diabetes mellitus), and 2041 kcal/24 h (obese with diabetes mellitus). Thus, BCAA concentrations seem to be affected not only by degree of obesity, but also by many confounding factors such as gender, age, energy expenditure, and analyzing method, which should be properly controlled.

*Aromatic Amino Acids*. Aromatic amino acids (AAAs) refer to amino acids that include an aromatic ring, such as phenylalanine, tyrosine, and tryptophan. Some are derived from diet, while others can be synthesis by human body. Recent studies showed AAAs were close to IR in obese adults and children, especially for tyrosine and phenylalanine. Butte et al. [21] found that tyrosine was the highest-ranked metabolite on the basis of its contribution to the obesity classification, with predictive accuracy as 81%. In their study, the AAAs (phenylalanine, tyrosine) together with BCAAs constitute PC6, which presented striking close relationship with IR. Consistent results were also found in studies by Perng et al. [23], Lee et al. [22], and Mccormack et al. [26]. Interestingly, most studies combine BCAAs and AAAs as a PC factor and analyze them together, and BCAAs and AAAs usually showed similar change tendency in obese children. The explanation of this consistency might be that AAAs can compete with BCAAs for uptake into tissues via their common neutral amino acid transporters. In obese people, the limited quantity of neutral amino acid transporters could not afford the excess BCAAs and AAAs transportation leading to their accumulation in bloodstream with high concentration. For a long time, LAT1 was believed to work as their transporters in broad tissues [36, 37]; however, recent studies have found the functional LAT1 protein only expressed in BBB and placenta [38]. There is no direct evidence that LAT1 protein exists on the plasma membrane of other tissues, which has been proved by the LAT1 specific PET probe ^18^F-FAMT [39–41]. Therefore, the competition of amino acids might occur at the other broad scope neutral amino acid transporters, which should be the major contributors and require further studies to validate.

Different from above results, a latest study from Hellmuth et al. [20] suggested tyrosine is the only metabolite which was significantly associated with HOMA-IR at baseline and after intervention in obese children. They thought tyrosine was the primary alteration and then resulted in BCAAs' elevation and development of IR. While these findings need more studies to be validated, further prospective, longitudinal studies are required to unravel associations between AAAs and IR.

*Other Amino Acids.* Besides BCAAs and AAAs, there were other amino acids related to IR, including sulfur amino acids (cysteine, homocysteine) [29], asparagine [21], glycine [21], serine [21], proline [20], citrulline [26], and glutamate [21, 24]. As for variation tendency of these metabolites, obese children usually presented elevated glutamate, proline, cysteine and decreased asparagine, glycine, citrulline, and serine when comparing with nonobese children.

#### 4.1.2. Metabolomics in Lipid Metabolism

It is well-established that diabetes is often accompanied by dyslipidemia [42], which is a major risk factor of cardiovascular diseases in diabetic patients [16]. More and more evidence suggests that increased free fatty acids and IR are the main causes of dyslipidemia [43, 44]. Thus, specific metabolites related to above pathways can provide reliable information about lipid metabolism in obese insulin resistant children.

*Fatty Acid Oxidation and Acylcarnitines.* Metabolomic profiles in obese people were reported to present distinct pattern on fatty acid oxidation and acylcarnitines [20, 22, 23]. Acylcarnitines are the by-products of noncomplete fatty acid oxidation [45]. Accumulation of fatty acid oxidation related metabolites, known as lipotoxicity, has been implicated in the development of IR and type 2 diabetes [17, 46, 47]. In addition, Newbern et al. [24] reported by-products of fatty acid oxidation C2 acylcarnitine divided by the sum of C3 and C5 inversely correlated with HOMA-IR. The negative correlation was also found in long-chain acylcarnitines and HOMA-IR in Newbern et al.'s study. Perng and his colleague found C3 acylcarnitine and C5 acylcarnitine were positively related to HOMA-IR [23]. Besides, Hellmuth et al. [20] used different ratios as biomarker for IR, such as C5/C6-oxo, C4/C5-oxo, C6-oxo/xLeu, and C5-OH/C5:1 in different subgroups. All of these indicated incomplete fatty acid oxidation was related to higher score of HOMA-IR. The possible mechanism might be reduced complete fatty acids oxidation can result in proinflammatory pathways simulation, disturbed insulin action in skeletal muscle, enhanced mitochondrial stress, and final dysglycemia in humans and rodents [45, 47, 48]. Butte et al. found significantly reduced lysolipids (glycerophosphocholines, glycerophosphoethanolamines) and dicarboxylated fatty acids in obese adolescents comparing with their counterparts [21, 49]. As for nonesterified fatty acids (NEFAs), Butte et al.'s and Newbern et al.'s studies suggested obese children showed increased NEFAs comparing with nonobese children. Taken together, potential important role of fatty acids oxidation might be involved in the development of IR and other metabolic disorders. Among alterations in metabolic pathway of lipids, C3 and C5 acylcarnitine were the most frequently mentioned acylcarnitine. Furthermore, because C3 and C5 acylcarnitines were the by-products of BCAAs, reduced complete fatty acids oxidation seemed to be influenced by BCAAs metabolism indicating close interaction of amino acid metabolism and lipid metabolism.

*Androgen Hormones.* Different from adults, adolescents experiencing pubertal growth might present alterations in metabolic hormones and body fat deposition. An elevated androgen derivative in obese group was reported by Butte et al. [21] and Perng et al. [23]. Perng et al. found children with higher BMI had a higher score for the sex steroids, HOMA-IR, and fasting insulin level. The increase of sex steroids might stimulate premature adrenarche [50] and induce the insulin-like growth factor/growth hormone (IGF/GH) axis [51], which is associated with many metabolic disorders including dyslipidemia, hyperinsulinism, metabolic syndrome, and polycystic ovary syndrome [52, 53], and this change was related to amino acid metabolism. No relationship was observed between pubertal status (before puberty and during puberty) and fasting BCAA levels although alterations in proteolysis and protein oxidation might exist [26, 54, 55]. Thus, although pubertal growth is related to specific sex steroid pattern and IR in obese children, it seems nonrelevant to altered amino acids metabolism.

### 4.2. Metabolomics as Biomarker for Future Metabolic Risk in Obese Children

An increasing number of studies have shown that the substantial susceptibility of diabetes acquired in youth indicated the importance of whole-life prevention and management [10]. However, a large part of adolescents with type 2 diabetes related obesity is undiagnosed [56]. Moreover, the undiagnosed type 2 diabetes in early life will progress with time passing and affect more and more pregnant women [57, 58]. Thus, it becomes especially crucial to perform effective prevention to early childhood. A useful and predictive biomarker for development of IR, risk of type 2 diabetes, and related metabolic disorders seems so significant nowadays, while existing studies on metabolomics and future metabolic risk were largely lacking and results were not quite consistent.

A recent study of Mccormack et al. [26] on 69 healthy children observed there was no association between elevated BCAAs and HOMA-IR at the time of recruitment, but higher BCAAs at baseline could predict worsening of IR after 18 months of follow-up among 17 participants with complete data. Of these 17 individuals followed in the longitudinal cohort, none had impaired fasting glucose (IFG) or impaired glucose tolerance (IGT) at baseline. 18 months later, none developed diabetes mellitus but five developed impaired fasting glucose and/or impaired glucose tolerance. Another study by Lee et al. [22] with a larger sample of 109 boys confirmed that BCAA concentrations higher than the median value could be an early biomarker of development of IR and related metabolic syndrome. Among the variable metabolites, only BCAAs showed a significant risk relationship with high HOMA-IR (>3.11) at 2-year follow-up after adjustment for baseline age and *z* scores of BMI as well as waist circumference (OR = 3.139, *p* = 0.042). For future metabolic risk score, BCAAs showed a marginally significant effect on metabolic syndrome risk (OR = 3.222, *p* = 0.066), while different results were from Hellmuth et al.'s study, which suggested tyrosine, rather than BCAAs, was the useful biomarker for future risk of IR and metabolic risk in 80 obese children with one-year follow-up. They found only tyrosine was significantly associated with HOMA (*p* < 0.05) at baseline and end of intervention. Despite controversial results, the available evidence still reflects the significant role of amino acid metabolism in the prediction of IR development and future health crisis. Moreover, it can also suggest that alterations in amino acids (BCAAs and tyrosine) might exist earlier than IR, manifesting the possible causal relationship between them to some extent. However, more large-scale, long-term studies in this field are extremely lacking and urgent.

### 4.3. Comparison on Metabolomics Indicators of IR between Obese Children and Adults

Refer to the different metabolomic signature in obese adults and children; the variances in manifestation of IR or obesity between them should be discussed at first. Due to the distinct developmental characteristics, children continue to grow with obvious tissue expansion and maturation [59]. It can also suggest the difference in capacity of metabolic adaption to obesity, also called metabolic flexibility [59, 60]. Comparing with adults, one striking difference in childhood obesity is that impairment of fasting glucose levels is usually absent and if present, it is a delayed finding. In fact, a definite increase in fasting or postprandial insulin can be identified as the very initial step in obese children.

Although metabolomic study is still in its infancy, a large body of evidence has emerged in recent years, showing the close relationship between IR and metabolomic profiles in obese adults [61–65]. Findings on obese children presented general consistency and promising expectation based on limited evidence. However, comparing with research in obese children, the studies in obese adults were more extensive and elaborate and with longer follow-up duration. The differences in metabolomic signature of IR between obese adults and children can be summarized into three aspects: affected metabolic pathways, accuracy in prediction of IR, and study design.

*Differences in Affected Metabolic Pathways of IR.* In present study, we found the most frequently affected metabolic pathways related to IR in obese children were amino acid metabolism and lipid metabolism. Besides above two important pathways, more derangements were revealed in adults with obesity and IR, such as glucose metabolism [66, 67], TCA cycle [67], inflammation [68], and gut microbiota [69]. Although some studies on obese children presented higher grade of inflammation compared with nonobese children, few studies showed the obvious relationship with statistic difference. As for the reason of inconsistency in glucose metabolism and TCA cycle between obese adults and children, it might be explained by the difference in manifestation of IR. In children, hyperinsulinemia was presented earlier than impairment in glucose metabolism and TCA cycle, which is contrary to adults. Gut microbiota has gained much more speculations in metabolic disorders of the host in the past decades [70, 71], especially in IR. Recently, metabolomic profiles from gut microbiota [69] showed significant differences in obese adults with IR compared to nonobese adults. However, little evidence was available on metabolomics from gut microbiota in obese children. Thus, future study should explore the significant role of metabolomics from gut microbiota in insulin resistant children.

In spite of the general accordance in amino acid metabolism and lipid metabolism, studies on adults were more widespread with larger amount of metabolites. The striking predictive values of BCAAs and AAAs in IR were accordant in adults and children, which might be the result of the increased proteolysis and impaired amino acid catabolism [72, 73]. It also indicates the significant role of amino acid in the pathology of IR. Besides these, adult studies showed their insights in glutamate, alanine, glycine, and so forth in amino acids metabolism and lysophospholipid, palmitic acid, intact acid, and so forth in lipid metabolism [74]. Studies on gut microbiota of adults presented significant changes in bile acids and choline [66, 75], calling for more research on children to validate these findings. Thus, studies on children should expand their research in a larger amount of people, in order to find undiscovered mechanism of IR.

*Differences in Predictive Power of IR.* To test the accuracy of metabolomic profiles in prediction of IR, ROC curves were applied in both obese adults and children studies. A study on adults showed that in total 399 subjects using metabolomics (a-hydroxybutyrate) to diagnose IR, 164 subjects were classified as insulin resistant and 235 subjects were classified as insulin sensitive [76, 77]. These results indicate a sensitivity of 85%, a specificity of 91%, and an overall prediction accuracy of 76%, while in Korean children with obesity [22] applying plasma BCAA to predict IR (HOMA-IR), the area under the curve (AUC) was 70.3% with the sensitivity and specificity as 74.1% and 58%, respectively. As for the metabolic risk score, AUC was 73%; the sensitivity and specificity were 77.8% and 59.3%, respectively. Thus, we can conclude that metabolomics can detect IR with high accuracy in both adults and children, although they did not use the same metabolites. However, few studies did this analysis which indicates future studies should add this part to the systematic evaluation of application for metabolomics. Also, using the same metabolites to compare difference in predictive power is required.

*Differences in Study Design*. Differences in study design between studies on adults and children involve three parts: the testing samples, sample size, and follow-up duration. For testing samples, available studies on obese children mainly target serum or plasma which means they can only test the changes of metabolites in the peripheral circulation. However, metabolomics approach can detect metabolites not only in blood, but also in urine and target tissues. Notably, testing metabolites in target tissues provides clues about more specific metabolic pathways affected in the tissue which can be validated at the gene level. Also, it provides more helpful information at the same time, which enables us to understand the role of target tissue in the changed metabolites level in the circulation. Comparing with children, studies on adults seem to be more diverse covering all three locations, including blood, urine, and tissues. And among various tissues, adipose tissue in adults showed significant role in plasma BCAA levels. In study of Badoud et al. [78], there are significant changes in BCAA catabolism and TCA cycle between obese people and lean people based on gene expression analysis of subcutaneous adipose tissue (SAT). Similar results were also shown by other studies based on omental adipose tissue and visceral adipose tissue [79, 80]. To find the gene variants in weight-discordant monozygotic twins, Naukkarinen and his colleagues [81] checked the SAT of obese cotwins and presented the downregulation of BCAA catabolism pathway, oxidative phosphorylation pathway, and fatty acid *β* oxidation. Future children studies should focus on genetic-metabolomic-phenotypic regulation of IR, which will offer more insights into the way of understanding metabolic disorders. As for the sample size and follow-up duration, studies on adults showed absolute advantage with larger sample size and longer follow-up duration in various racial subjects.

Thus, future studies of obese children should focus on the standardization of study design in order to detect metabolomic profiles in multiple tissues in larger and different racial people with long-term visit.

### 4.4. Strength and Limitation of This Systematic Review

This is the first systematic review on metabolomics profiles and IR related metabolic disorders in childhood obesity. Strengths of this systemic review include the comprehensive review and critical evaluation of current available literature. All ten studies were published from 2012 to 2016, which manifested the latest development in this area. However, there are still some factors limiting the ability to unravel conclusions, such as the small sample sizes of studies and short term follow-up. In addition, we used HOMA-IR as surrogate markers of IR and metabolic disorders at both baseline and follow-up, rather than the standard methods for measuring IR, such as the hyperinsulinemic-euglycemic glucose clamp test and OGTT. Not only the heterogeneity in study design and experimental method, but also the difference in subjects can influence the final results to some extent. Thus, it is crucial to carry on some large, well-defined cohorts offering robust analysis and complete data in this field.

## 5. Conclusion

As childhood obesity has become a global public health burden, more effective and preventive strategies are required. Our systematic review showed that insulin resistance in obese children was associated with distinct metabolomic profiles. Two different metabolic pathways, amino acid metabolism and lipid metabolism, seemed to be mainly affected in obese children comparing with nonobese children. Among metabolites, BCAAs, aromatic amino acids, and acylcarnitines were closely related to IR and future metabolic risk. Due to existing studies' limitation, more longitudinal and large-scale studies are required.

## Figures and Tables

**Table 1:** Characteristics of study population, HOMA-IR, and identified metabolomic profiles in 10 included studies.

Study	Year	Num	Male	Type	Mean age	BMI *z* score	HOMA-IR	Plasma metabolomic profiles identified
Hellmuth et al. [20]	2016	80	45%	C	11.5 ± 2.4	Obese: 2.40 ± 0.45	Obese: 4.29 ± 3.10	Total aromatic amino acids, C0, C3, C6:1-DC, C6-oxo, C4/C5-oxo, C5/C6-oxo, C6:1-DC/C5:1, C6-oxo/xLeu, valine, tyrosine, proline, serine, phenylalanine, glycine, histidine, and methionine

Butte et al. [21]	2015	803	50%	O	11.1 ± 3.9	Obese: 2.31 ± 0.38; nonobese: 0.64 ± 0.76	Obese: 7.46 ± 5.47; nonobese: 3.01 ± 2.49	Leucine, isoleucine, valine, glutamate, alanine, aspartate, glycine, serine, threonine, histidine, PUFA, peptide, tyrosine, methionine, bradykinin, lysine, phenylalanine, pyroglutamine, polyamine, urea cycle, polypeptide, short-chain acylcarnitines, long-chain acylcarnitine, ketone bodies, dicarboxylated fatty acids, lysolipid, medium-chain fatty acid, monoacylglycerol, phospholipid, androsterone sulfate, epiandrosterone sulfate, steroid derivatives, mannose, pyruvate, glycerate, pantothenate and CoA, citrate, purine, xanthine, pyrimidine, 2-methylbutyrylcarnitine, 3-methyl-2-oxobutyrate, and isovalerylcarnitine

Lee et al. [22]	2015	109	100%	C	10.4 ± 0.6	Obese: 1.49 ± 0.39; nonobese: −0.01 ± 0.15	Obese 2.30 ± 2.30; nonobese: 1.20 ± 0.60	Leucine, isoleucine, valine, phenylalanine, tyrosine, glutamine, alanine, glycine, serine, asparagine, lysine, glutamate, proline, citrulline, alpha-aminoadipic acid, C0, C3, C5, C7-DC, C16, and C18

Perng et al. [23]	2014	262	50%	O	8.0 ± 0.9	Obese: 2.07 ± 0.29; nonobese: −0.04 ± 0.72	Obese: 3.49 ± 1.80; nonobese: 1.65 ± 0.65	Leucine, isoleucine, valine, phenylalanine, C3, C5, tryptophan, 3-methyl-2-oxovalerate, kynurenine, tyrosine, *γ*-glutamylleucine, 4-methyl-2-oxopentanoate, isovalerylcarnitine, isobutyrylcarnitine, DHEA-S, 4-androsten-3beta, 17beta-diol disulfate, epiandrosterone sulfate, androsterone sulfate, pregn steroid monosulfate, pregnen-diol disulfate, pregnenolone sulfate, and andro steroid monosulfate

Newbern et al. [24]	2014	82	50%	O	13.8 ± 0.2	Obese male: 2.37 ± 0.06; obese female 2.24 ± 0.05	Obese male: 4.08 ± 0.48; obese female: 3.56 ± 0.27	Valine, leucine/isoleucine, glutamate/glutamine, C2, C3, C5, total BCAA, *β*-hydroxybutyrate, C2 to (C3 + C5) ratio, uric acid, histidine, C5-OH/C3-DC, C8:1, C4-DC/Ci4-DC, C10:2, C16, glycine, serine, tyrosine, phenylalanine, methionine, proline, alanine, ornament, arginine, C18:1, C18, and C18:1-OH/C16:1-DC

Farook et al. [25]	2015	42	45%	O	11.5 ± 3.1	BMI, obese: 27.5 ± 4.1; overweight: 23.0 ± 3.8; nonobese: 18.1 ± 2.8	Obese: 2.1 ± 0.5; overweight: 1.8 ± 0.4; nonobese: 1.5 ± 0.6	L-Thyronine, bradykinin, indole-3-propionic acid, naringenin, 2-methylbutyroylcarnitine, 3-hydroxyquinine, LysoPC (18:1), vitamin D3, calicoferol B, diglyceride, malvidin 3-(6-acetyl glucoside), linoleic acid, phosphatidylethanolamine, and phosphocholine (16:1)

Mccormack et al. [26]	2013	69	58%	C	13.3 ± 2.9	1.04 ± 1.23 Cohort: 2.88 ± 2.17	1.84 ± 2.04 Cohort: 1.50 ± 0.87	Valine, leucine, isoleucine, citrulline, glutamate, 3-hydroxyanthranilic acid, and phenylalanine

Michaliszyn et al. [27]	2012	139	47%	O	13.4 ± 0.2	BMI, obese: 32.50 ± 0.90; nonobese: 18.90 ± 0.30	Obese: 8.01 ± 1.90; nonobese: 4.58 ± 1.50	Valine, leucine/isoleucine, methionine, phenylalanine, glycine, serine, histidine, arginine, C2, C3, C5, C4, tyrosine, alanine, and citrulline

Mihalik et al. [28]	2012	103	52%	O	13.3 ± 0.2	BMI, obese: 34.60 ± 0.70; nonobese: 19.00 ± 0.30	Obese: 8.10 ± 3.20; nonobese: 4.00 ± 2.10	Leucine/isoleucine, valine, phenylalanine, methionine, glycine, serine, histidine, arginine, alanine, citrulline, C2, C3, C4, C4-OH, C5, C5:1, C5-OH, C6, C6-OH, C8, C8:1, C10, C10:1, C10:2, C12, C12-OH, C12:1, C14, C14:1, C14:2, C16, C16:1, C16-OH, C18, C18:1, C18:1-OH-CN, C18:2, and free CN

Elshorbagy et al. [29]	2012	984	50%	O	11.0 ± 2.2	BMI, obese: 27.3 ± 3.73; nonobese: 17.51 ± 1.47	Obese: 4.84 ± 2.26; nonobese: 2.16 ± 0.78	Total homocysteine, total cysteine, total glutathione, nonesterified fatty acids, and methionine

Note: num: number; O: observational study; C: cohort study; BMI*z*: body mass index *z* score; HOMA-IR: homeostasis model assessment of insulin resistance; C0: C0 acylcarnitine; C2: C2 acylcarnitine; C3: C3 acylcarnitine; C4: C4 acylcarnitine; C5: C5 acylcarnitine; C7: C7 acylcarnitine; C16: C16 acylcarnitine; C18: C18 acylcarnitine; PUFA: polyunsaturated fatty acids.

**Table 2:** Specific metabolites identified to be related to insulin resistance and future metabolic risk based on 10 included studies.

Affected metabolic pathways		Metabolites related to IR	Reference
Amino acids metabolism	Branched-chain amino acids	Valine, leucine, and isoleucine	[21–24, 26]
		Isovalerylcarnitine and isobutyrylcarnitine	[21, 23]
	Aromatic amino acids	Tyrosine and phenylalanine	[20–23, 26]
	Sulfur amino acids	Cysteine and homocysteine	[29]
	Other	Asparagine, glycine, serine, proline, citrulline, glutamate, and methionine	[20, 21, 24, 26]

Lipid metabolism	Acylcarnitines	C2, C3, C5, C2/(C3 + C5), C5/C6-oxo, C4/C5-oxo, C6-oxo/xLeu, and long-chain dicarboxylic acylcarnitines	[20, 22–24]
	Fatty acids	NEFA and FAO by-products	[21, 24]
	Steroid	Androgen hormones	[21, 23]
